# A Simple and Accurate Method of Standardizing Vaccines

**Published:** 1913-12

**Authors:** 


A Simple and Accurate Method of Standardizing Vaccines. Ward Burdick, M. D., Denver (*Colorado Med.*) A solution consisting of distilled water nine parts, and Loeffler's alkaline methylene blue one part, is kept on hand. Preparatory to standardizing an emulsion, a piece of quarter-inch glass tubing is drawn out in the flame, as in making capillary pipettes for blood serum work. The glass is now scratched with a file midway on the capillary portion and carefully severed into two capillary pipettes, each obviously having a capillary aperture of exactly equal diameter. Into a watch crystal are placed, say, nine drops of filtered methylene blue solution with one of the pipettes, held in a vertical position, with just enough pressure exerted on the teat to promote the collection of the drop at the point of the pipette. To this is now added one drop of bacterial emulsion, with the other fresh pipette, held in the same manner. The drops from each pipette will be of equal size, since the capillary apertures are of the same diameter and both manipulated in the same manner. One has now in the watch crystal an exact 1-10 dilution of the bacterial emulsion; this is now allowed to stand ten or fifteen minutes, during which time the germs will become stained. With a fresh pipette the dilution is now carefully drawn up and discharged into the dish several times to insure equal distribution of germs in the mixture. With the same pipette a suitable drop of the dilution is now placed on the island of an ordinary blood counting chamber covered with a thin cover glass, and set aside for a few moments until the germs have settled on the ruled surface, where they may be counted in the same manner as are red blood corpuscles and where they will be plainly visible by reason of the methylene blue taken up by them. The degree of dilution may be varied to suit the density of the bacterial emulsion to be standardized.

After this procedure there are no expensive pipettes to be cleaned and sterilized with difficulty. Those used in this method are dropped into the autoclave to be run up with the next steaming and discarded. The counting chamber is placed in cold lysol solution and given sufficient exposure to destroy the germs being handled, after which it is held under the cold water tap, thoroughly rinsed in running water and dried with a cotton cloth. The watch crystal is treated in the same way and dropped into cleaning fluid, where it remains until again brought into use.

